# Blood Lead Levels in Children Living Near an Informal Lead Battery Recycling Workshop in Patna, Bihar

**DOI:** 10.5696/2156-9614-10.25.200308

**Published:** 2020-02-28

**Authors:** Jamal Akhtar Ansari, Abbas Ali Mahdi, Promila Sharma Malik, Tabrez Jafar

**Affiliations:** 1 Department of Biochemistry, King George's Medical University, Lucknow, India; 2 Department of Chemistry, Shibli National PG College, Azamgarh, India; 3 Era University, Lucknow, India; 4 Pure Earth, India Office, New Delhi, India

**Keywords:** lead toxicity, Patna, Bihar, informal lead battery recycling

## Abstract

**Background.:**

Lead can cause significant biological and neurologic damage, even at small concentrations, and young children are at higher risk. Informal recycling of lead batteries and lead-based workshops/industries have increased the burden of lead toxicity in developing countries, including India. Many informal recycling lead battery workshops have been established by the local people of Patna, Bihar as self-employment opportunities. However, most of the residents are not aware of the risk factors associated with lead poisoning.

**Objectives.:**

The present pilot study aimed to assess blood lead levels (BLLs) and hemoglobin levels among children aged between 3 to 12 years in the settlement of Karmalichak near Patna, India.

**Materials and Methods.:**

Children residing near the informal lead battery manufacturing unit were selected for BLL assessment. A total of 41 children were enrolled in the questionnairebased survey.

**Results.:**

All the children in the present study had detectable lead concentrations in their blood. Only 9% of the studied children had a BLL ≤5 μg/dl, while 91% children had a BLL above >5 μg/dl.

**Conclusions.:**

The present study carried out in children of Karmalichak region of Patna, India was an attempt to better understand the problem of lead toxicity, describe the epidemiology of its adverse effects, identify sources and routes of exposure, illustrate the clinical effects and develop strategies of prevention so that remedial measures may be taken by government agencies and regulatory bodies. In view of the high lead levels in children in the study area, attempts are being made to develop strategies for future prevention by relocating the informal battery recycling workshops from the area. Moreover, parents have been advised to increase nutritional supplementation of children by providing calcium-, iron- and zinc-rich foods, including milk and vegetables.

**Participant Consent.:**

Obtained

**Ethical Approval.:**

The study was approved by the ethical committee of Era's Lucknow Medical College & Hospital, Era University, Lucknow (India).

**Competing Interests.:**

The authors declare no competing financial interests.

## Introduction

The Centers for Disease Control and Prevention (CDC) declared that lead poisoning is one of the most common and preventable pediatric health problems today.^1^ Even at low levels, lead exposure in children causes reduction in IQ and attention span, reading and learning disabilities, hyperactivity, impaired growth, behavior problems and hearing loss. These effects are long term and may be irreversible.^2^,^3^

Lead poisoning is not only associated with abnormal hemoglobinization of erythrocytes, but also negatively affects red blood cell production. Lead exposure does not invariably lead to anemia in adults, however, in children it may be marked.^4–6^ The World Health Organization (WHO) estimates that the highest rates of mild mental retardation caused by exposure to lead occur in developing countries, where mean blood-lead concentrations are typically higher than those in developed countries.^7^ In India, it has been reported that nearly half of the child population has elevated blood lead levels (BLLs).^8^

In India, lead poisoning has increasingly become an issue due to rapid industrial expansion in the last century and unregulated use of lead-based materials. In addition to the common uses of lead in paint, firearms, battery recycling and automobile repair, lead is also used in herbal and traditional medicines in India.^9^ In addition, there are no uniform policies in India regarding routine assessments of heavy metals, especially lead in soil and drinking water. However, recently, the Ministry of Environment, Forest and Climate Change, Government of India passed a notification in November 2016 titled the “Regulation on Lead Contents in Household and Decorative Paints Rules, 2016” which prohibits the manufacture, trade, import or export of household and decorative paints containing lead or lead compounds in excess of 90 ppm.^10^ The informal recycling of automobile batteries and unsystematic regulation of lead battery workshops/industries has increased the burden of lead toxicity in developing countries, particularly in India.^11^ Many battery recycling workshops and smelters have been established in local areas of Patna, Bihar. This has provided employment opportunities for local members of the community. However, concerns remain regarding appropriate regulation of the smelter and possible ongoing lead exposure in the environment. Two rounds of environmental assessments and soil test results in 2015 and 2017 showed elevated levels of lead in soil around the manufacturing sites.

In response, a global environmental health non-governmental organization, InSLAR (Indian Society for Lead Awareness and Research, www.inslar.org), together with several local partners, coordinated the present study, a health assessment of high-risk populations in Karmalichak, near Patna, Bihar in February 2018. The objective of the present study was to determine BLLs and hemoglobin levels of children aged between 3 to 12 years in the Karmalichak region in Patna, India, as well as to quantify the problem and provide a preliminary assessment of lead poisoning prevalent in the area.

**Abbreviations**
*BLLs*: Blood Lead Levels

## Materials and Methods

Karmalichak is a peri-urban, small and poor neighborhood in the suburbs, about 14 km from Patna city, in Bihar State, India. The site is a single battery plate manufacturer, reportedly operating illegally over the last decade. Two rounds of environmental assessments and soil test results in 2015 and 2017 showed elevated levels of lead in soil around the manufacturing site.^12^ The immediate vicinity is residential with a high population density, with roughly 230,000 people residing in the area as per the report of the Government of Bihar (India).^13^ A private primary school with 250 enrolled children, mostly under 12 years of age, is located opposite the informal battery recycling manufacturing unit.

### Sampling

Informed, written consent was given by the parents of the children taking part in the present study. The questionnaire form included socio-demographic questions, including age, smoking habits, family member smoking, water source, residential location, occupation, family members working in lead-exposed professions, pica symptoms, previous history of lead exposure, drinking habits, and presence of any other self-reported disease indicating possible lead exposures *(Supplemental Material).* Children residing near the informal lead battery manufacturing unit were randomly selected for BLL assessment. A simple random sampling of 41 children was performed out of 100 children enrolled for the study using a lottery method, as this was a pilot study. Sample size was calculated on the basis of variation in BLL among the study participants using the formula for estimation of mean discussed in ‘A. Indrayan, Basic Methods of Medical Research’ using the half IQR of BLL (5.45) as an estimate of variablity and considering 0.25-times mean BLL (12.0) as clinically significant difference.^14^ Considering a 95% confidence level and 90% power of study, the minimum sample size was calculated to be 41. Randomization was performed using a computer-generated random number table by taking 41 random numbers from a table of random numbers from 1 to 100. Subjects were selected from the sequence of 100 enrolled subjects whose serial numbers were matched with the generated random numbers.

About 4–5 ml of venous blood sample was collected from each subject in an ethylenediaminetetraacetic acid vial. The study was approved by the Ethics Committee of Era's Lucknow Medical College & Hospital, Era University, Lucknow (India).

### Blood lead level estimation

A LeadCare II (Meridian Bioscience, Cincinnati, OH, USA) analyzer using an electrochemical technique called anodic stripping voltammetry was used to determine the amount of lead in the blood samples.^15^ The LeadCare II system relies on electrochemistry and has a unique sensor to detect lead (detectable range 3.2–60 μg/dl) in whole blood. When whole blood is mixed with the treatment reagent, the red blood cells are lysed, and the lead is made available for detection. When a test is run, the analyzer applies a potential that causes the lead to collect on the LeadCare II sensor. After three minutes, the analyzer measures the amount of lead collected on the sensor and display the result in μg/dl.

#### Hemoglobin level

As anemia is common in children exposed to lead, we assessed the hemoglobin levels of all of the children in the present study. Hemoglobin estimation was done with Sahli's hemoglobinometer using the acid hematin method.^16^ Blood was mixed with N/10 hydrogen chloride, which resulted in converting hemoglobin to a brown-colored acid hematin. Thereafter, the solution was diluted until the color matched with the color of the comparator box. The concentration of hemoglobin was reported in gm/dl.

## Results

In the present study, the lowest value BLL was 9.1 μg/dl, and the highest BLL value recorded was 80.1 μg/dl in children. Across the samples, hemoglobin levels ranged from 7.8 gm/dl to 12.0 gm/dl in children with a high BLL (>5 μg/dl) *(Table 1).*

**Table 1:** Blood Lead and Hemoglobin Levels of Children in Karmalichak, Patna, Bihar

**Age (years)**	**BLL (μg/dl)**	**Hemoglobin level (mg/dl)**
4	9.4	9.1
4	10.2	11.1
4	12.3	9.1
4	25.0	10.3
5	ND	13.9
5	13.4	8.6
5	21.7	9.4
5	58.5	8.0
6	14.7	11.1
6	24.1	9.6
6	26.8	9.2
6	51.2	8.1
7	10	10.4
7	13.9	10.7
7	17.5	9.4
7	17.9	10.1
7	19.2	9.1
7	21.7	10.3
7	33.4	8.9
7	40.3	7.9
7	80.1	7.9
8	17.3	10.1
8	24.4	10.7
8	26.0	8.6
8	31.8	9.3
8	32.0	8.9
8	37.4	8.7
9	9.3	12.0
9	10.7	11.7
9	17.9	11.4
9	32.4	8.9
10	ND	14.7
10	25.1	9.7
11	ND	14.1
11	ND	15.0
11	9.1	11.8
11	13.9	10.7
11	18.9	9.7
11	75.6	7.8
12	26.1	10.8
12	72.9	8.3

Abbreviation: ND, not detected.

### Child blood lead levels

The average age of subjects was 7 years. The mean BLL across all ages was 24.4 μg/dl, with a median of 19.2 μg/dl (SD 19.46).

In the present study, 23.5% of the children in the age group of 0–5 years had BLL of >5–20 μg/dl, while 15% had BLL >20 μg/dl. In addition, 52.9% of children between 5–8 years old had BLL >5–20 μg/dl and 55.0% had >20 μg/dl. Moreover, 23.5% of the children between 8–12 years had BLL >5–20 μg/dl and 30.0% had BLLs >20 μg/dl. In the group with BLL >5–20 μg/dl, 41.1% cases were male and 58.8% were female. In the group with BLL >20 μg/dl, 65.0% were male subjects and 35.0% were females *(Table 2).*

**Table 2:** Factors Associated with Blood Lead Levels Among Children in Karmalichak, Patna

**Characteristics**	**≤5 μg/dl (n=4) n (%)**	**>5–20 μg/dl (n=17) n (%)**	**>20 μg/dl (n=20) n (%)**
**Child demographics**			
*Age*			
(0–5)	1 (25.0)	4 (23.5)	3 (15)
(5–8)	0(0)	9 (52.9)	11 (55.0)
(8–12)	3 (75.0)	4 (23.5)	6 (30.0)
*Gender*			
Male	3 (75.0)	7 (41.1)	13 (65.0)
Female	1 (25.0)	10 (58.8)	7 (35.0)
*Child attributes*			
Thumb sucking	0 (0)	0 (0)	0 (0)
Handwashing before eating	4 (100)	17 (100)	20 (100)
Pets in house	2 (4.8)	5 (29.4)	4 (20.0)
Colored toys at home	0 (0)	10 (58.8)	11 (55.0)
Eating food from roadside vendors	2 (50.0)	16 (94.1)	18 (90.0)
Using kohl	1 (25.0)	8 (47.0)	9 (45.0)
Going to school	4 (100)	16 (94.1)	20 (100)
Use of Ayurvedic/folk medicine	0 (0)	0 (0)	0 (0)
**Maternal attributes**			
Cosmetic use of kohl/surma/sindoor/lipstick/dye	4 (100)	17 (100)	20 (100)
**Paternal attributes**			
*Occupation* Farmers	4 (100)	17 (100)	20 (100)
*Demographic attributes*			
Municipal water supply w/o water purification	4 (100)	6 (35.2)	8 (47.0)
Utensils – steel/aluminum/ceramic	4 (100)	17 (100)	20 (100)
**Fuel**			
Kerosene	0 (0)	0 (0)	0 (0)
Liquefied petroleum gas (cooking gas)	4 (100)	17 (100)	20 (100)
Coal	0 (0)	0 (0)	0 (0)
Wood	0 (0)	0 (0)	0 (0)
**Housing**			
Housing > 10 years old	4 (100)	10 (58.8)	16 (94.1)
Recent paint in home (0–5 years)	2 (50)	13 (76.4)	11 (64.7)
Recent plumbing in home (0–1) years	1 (25)	3 (17.6)	2 (10)
Distance from traffic congestion (0–1.0 km)	4 (100)	17 (100)	20 (100)

### Hemoglobin levels

Hemoglobin levels were found to decrease in subjects with elevated BLLs. The mean value of hemoglobin was found to be 10.1 gm/dL±1.87. Low hemoglobin levels (7.9 gm/dl) were also observed in a child with the highest BLL (80.1 μg/dl).

### Demographic attributes

The demographic evaluation of data *(Table 2)* revealed that 100%, 35.2% and 47% of children with BLLs of ≤5 μg/dl, >5–20 μg/dl, and >20 μg/dl, respectively, were in families using the municipal water supply without any water purification system. The rest of the families used water purification systems. Furthermore, 58.5% of the children associated with BLLs >5–20 μg/dl and 94.1% of children associated with >20 μg/dl lived in homes which were >10 years old.

#### Physical symptoms (as reported by parents/guardians/children)

It was reported that most of the children with BLL >5 μg/dl struggled with problems of hyperactivity, deteriorating academic performance, tiredness, headache, lethargy, abdominal pain, etc. These symptoms were greater in children with high BLLs *(Table 3).*

**Table 3:** Clinical features (as reported by parents/guardians/children)

**Clinical features**	**≤5 μg/dl (n=4) n (%)**	**>5–20 μg/dl (n=17) n (%)**	**>20 μg/dl (n=20) n (%)**
Tiredness	0 (0)	3 (17.6)	8 (40)
Headache	0 (0)	1 (5.8)	5 (25)
Lethargy	0 (0)	2 (11.7)	3 (15)
Gastro-intestinal, anorexia	0 (0)	2 (11.7)	6 (30)
Constipation	0 (0)	1 (5.8)	2 (10)
Pain abdomen	0 (0)	2 (11.7)	3 (15)
Hyperactivity	1 (25)	4 (23.5)	6 (30)
Deteriorating academic performance	1 (25)	5 (29.4)	6 (30)
Sleeping problems	0 (0)	3 (17.6)	3 (15)
Delirium	0 (0)	1 (5.8)	2 (10)
Convulsions	0 (0)	0 (0)	0 (0)

## Discussion

The present study found that approximately 90% of tested children in Karmalichak, Patna had BLLs above 5 μg/dl. Moreover, in five children, BLLs were found to be greater than 45 μg/dl, indicating a possible need for chelation therapy. Although the present study did not test for cognitive and behavioral problems related to lead poisoning, deteriorating academic performance, delirium and irritability were reported by some of the children and their parents. Other clinical symptoms, such as abdominal pain, tiredness, headache, and lethargy were also reported by some children. This may be non-specific to lead poisoning, however.

Elevated lead levels had been previously found in the soil.^12^ Close proximity of residential homes to the lead recycling workshop may be a risk factor for elevated soil lead content.^12^,^17^

Elevated BLLs have been documented in children exposed to industrial emissions from battery recycling workshops and smelting activities.^18–21^ Lead pollution is a public health concern in developed, as well as developing, countries. There have been reports that over half of the children in India have elevated BLLs (>5 μg/dl).^22^,^23^ Moreover, the National Health Family Survey has shown elevated BLLs in children <3 years old in two major cities of India, Mumbai and Delhi.^23^ In addition, over 12% of the children tested had BLLs greater than 20 μg/dL.^24^,^25^

Several studies across the globe have shown varying BLLs based on localization, industrialization, and exposure to lead. For example, Reuben *et al.* reported a mean BLL value of 10.99±4.63 μg/dL in the United States.^26^ Similarly, there are other reports of elevated BLLs from numerous countries, such as 8.6±2.8 μg/dL in Indonesia, 20.33±9.36 μg/dL in Nepal, 15 μg/dL in Bangladesh, 18.2 μg/dL in Bulgaria, 11 μg/dL in Romania, 18.9 μg/dL in Hungary and 7.4 μg/dL in Italy.^27–30^

The CDC states that primary prevention should be “a strategy that emphasizes the prevention of lead exposure, rather than a response to exposure after it has taken place”.^31^ There have been reports that nutritional deficiencies may increase the uptake of toxic elements, including lead.^6^,^32^ Nutritional supplements such as calcium, iron, zinc, vitamin C and protein may decrease absorption of ingested lead in children.^33–35^ Moreover, chelation therapy is recommended in subjects with a BLL >45 μg/dL. Succimer (dimercaptosuccinic acid) is an oral chelating agent and calcium disodium edentate (calcium disodium ethylenediaminetetraacetic acid) can be given by continuous intravenous infusion in high BLL cases.^35–38^

### Study limitations

Study limitations include the small sample size and that drinking water and soil were not analyzed for lead. Furthermore, health and habits were reported only by the head of household, typically fathers.

## Conclusions

The rising trend of lead poisoning in children is a cause for serious concern. The present study found that children in Karmalichak, Patna were exposed to lead from the informal recycling of lead batteries. Children with elevated BLLs in this community need to be followed up and corrective measures must be taken to reduce BLLs. Low income individuals are more likely to live in close proximity to workshop sites or engage in informal activities that expose them to lead, even within their own homes. These small informal workshops require better monitoring and enforcement of regulations. Future research should focus on identification of other risk factors, including drinking water and soil sampling within the vicinity and examination of the full burden of this preventable problem through large multi-centric epidemiological studies as well as the introduction of community awareness programs on lead poisoning.

In view of the high lead levels in children living in Karmalichak, Patna, India, attempts are being made to develop strategies for future prevention by relocating the informal battery recycling workshops from the area. In addition, government agencies and regulatory bodies are being urged to take remedial measures. Moreover, parents have been advised to increase nutritional supplementation of children by providing calcium-, iron- and zinc-rich foods, including milk and vegetables.
