# Supplementary material for: Blood Lead Levels in Children Living Near an Informal Lead Battery Recycling Workshop in Patna, Bihar
Source: J Health Pollut. 2020 Feb 28;10(25):200308. doi: 10.5696/2156-9614-10.25.200308 (PMC7058140; doi:10.5696/2156-9614-10.25.200308)
Supplement: Supplementary file 1 [file Ansari_Supplemental.doc]

| **CHILD PARTICULARS** |
| --- |
| Name: Age Sex Religion |
| Father’s name Address: Phone No. |
| **CHILD ATTRIBUTES** |
| **-**Thumb sucking Y/N |
| **-**Hand-washing before eating Y/N |
| **-**Pets in house present Y/N |
| **-** Colored toys at home present Y/N |
| **-** Whether eating stuffs from road side vendors Y/N |
| **-** Kohl use in child Y/N |
| **-** Whether going to school Y/N |
| - Whether child takes ayurvedic/ folk medicine Y/N |
| **MATERNAL ATTRIBUTES** |
| **-**Age |
| **-**Parity (No. of pregnancies) |
| **-**Education |
| **-**Cosmetics use like |
| kohl/ surma Y/N |
| Sindoor Y/N |
| Lipstick Y/N |
| Dye Y/N |
| **PATERNAL ATTRIBUTES** |
| -Age |
| -Education |
| -Occupation |
| car repair |
| cement/ construction |
| plastic manufacturing |
| ceramics |
| batteries |
| lock industries |
| polishing, soldering |
| furniture |
| radiation |
| farming |
| Any other |
| **DEMOGRAPHIC ATTRIBUTES** |
| Source of water supply Government supply/ handpump/ summersable |
| Utensils for eating |
| steel Y/N |
| Aluminium Y/N |
| Ceramic Y/N |
| Fuel |
| - kerosene Y/N |
| -Liquefied Petroleum Gas (cooking gas) Y/N |
| -coal Y/N |
| -wood Y/N |
| Housing |
| -how much old (months/years) |
| -recent paint (months/years) |
| -recent plumbing (months/years) |
| Distance of house from traffic congestion (in kms) |
| **CLINICAL SYMPTOMS** |
| Non specific - Tiredness Y/N |
| Headache Y/N |
| Lethargy Y/N |
| Gastro-intestinal – anorexia Y/N |
| Constipation Y/N |
| Pain abdomen Y/N |
| Neuro-behavioral- irritability Y/N |
| Hyperactivity Y/N |
| Deteriorating academic performance Y/N |
| Sleeping problems Y/N |
| Delirium Y/N |
| Convulsions Y/N |
| **ANTHROPOMETRIC/NUTRITIONAL PARAMETERS** |
| Nutritional Status: Veg/ Non-Veg/ Omnivorous |
| Malnourished: Y/N |
| Weight |
| Height |
| Pallor |
| BMI: |
| Lead line on gums (Burtonian lines) Y/N |
| **ANY PREVIOUS DISEASE** |
| Anemia Y/N |
| Malaria Y/N |
| Other Disease |
| Other issue regarding mental development etc. |
